# The Effects of Interacting With a Paro Robot After a Stressor in Patients With Psoriasis: A Randomised Pilot Study

**DOI:** 10.3389/fpsyg.2022.871295

**Published:** 2022-05-12

**Authors:** Mikaela Law, Paul Jarrett, Michel K. Nieuwoudt, Hannah Holtkamp, Cannon Giglio, Elizabeth Broadbent

**Affiliations:** ^1^Department of Psychological Medicine, The University of Auckland, Auckland, New Zealand; ^2^Department of Dermatology, Middlemore Hospital, Auckland, New Zealand; ^3^Department of Medicine, The University of Auckland, Auckland, New Zealand; ^4^The Photon Factory, The University of Auckland, Auckland, New Zealand; ^5^School of Chemical Sciences, The University of Auckland, Auckland, New Zealand; ^6^The MacDiarmid Institute for Advanced Materials and Nanotechnology, Wellington, New Zealand; ^7^The Dodd-Walls Centre for Photonic and Quantum Technologies, Dunedin, New Zealand

**Keywords:** psoriasis, psychological stress, robotics, Raman spectrum analysis, skin

## Abstract

**Objective:**

Stress can play a role in the onset and exacerbation of psoriasis. Psychological interventions to reduce stress have been shown to improve psychological and psoriasis-related outcomes. This pilot randomised study investigated the feasibility of a brief interaction with a Paro robot to reduce stress and improve skin parameters, after a stressor, in patients with psoriasis.

**Methods:**

Around 25 patients with psoriasis participated in a laboratory stress task, before being randomised to either interact with a Paro robot or sit quietly (control condition) for 30 min. Raman spectroscopy and trans-epidermal water loss were measured at baseline, after the stressor and after the intervention as indexes of acute skin changes. Psychological variables, including self-reported stress and affect, were also measured at the three time-points.

**Results:**

No statistically significant differences between the two conditions were found for any of the outcomes measured. However, effect sizes suggest significance could be possible with a larger sample size. Changes in the psychological and Raman spectroscopy outcomes across the experimental session were found, indicating the feasibility of the procedures.

**Conclusion:**

This pilot study showed that a brief interaction with a Paro robot was a feasible intervention for patients with psoriasis, but future trials should broaden the inclusion criteria to try to increase recruitment rates. Studying people who are highly stressed, depressed or who are stress-responders may increase the power of the intervention to show effects using a longer-term intervention.

## Introduction

Psoriasis is a common, chronic inflammatory skin condition that is characterised by red, scaly plaques that predominantly affect the elbows, knees, and scalp, with a worldwide prevalence of approximately 2%–3% (Gisondi et al., 2020; Parisi et al., 2020). The plaques develop due to a hyperproliferation of keratinocytes and upregulation of inflammatory cytokines in the skin (Albanesi et al., 2018). Patients with psoriasis have an accelerated rate of epidermal cell reproduction and therefore impaired skin barrier function (Maddock et al., 2019). Psoriasis may not be stable and patients can experience variability in its severity, with periods of remission or exacerbation through their life (O'Leary et al., 2004).

Patients with psoriasis may have substantial psychiatric comorbidities, which can significantly impair their quality of life. Psoriasis causes high levels of psychological distress and is associated with higher than normal rates of clinical depression and anxiety (Fortune et al., 2000; Kotrulja et al., 2010). Social embarrassment, isolation, rejection, and poor body image are also common, as psoriasis is a visible and therefore stigmatising condition (Bundy et al., 2013). This level of stigmatisation strongly affects mental health. Psoriasis can therefore negatively affect psychological, vocational, social, and physical functioning, making it imperative to improve the disease and symptoms to minimise these adverse effects (Shah and Bewley, 2014).

Although the aetiology of psoriasis is unclear, both genetic and environmental factors play a role (Heller et al., 2011). Both observational and experimental studies demonstrate that stress may play a critical role in the onset and exacerbation of psoriasis and stress can impede the success of treatments (Fortune et al., 2003; Verhoeven et al., 2009; Stewart et al., 2018). It is a cycle, as stress can worsen psoriasis and in turn, stress is also a consequence of psoriasis (Heller et al., 2011). This is particularly true for a subset of patients who believe that stress affects their psoriasis. These patients are termed stress responders and they have been found to have different physiological responses to stress that can in turn cause exacerbations (Zachariae et al., 2004; Fordham et al., 2013). Although the role of the hypothalamic–pituitary–adrenal (HPA) axis and cortisol has been implicated, the exact mechanisms for the relationship between psoriasis and stress are yet to be fully elucidated (Stewart et al., 2018; Yang and Zheng, 2020).

Psychotherapeutic treatments to reduce stress may be beneficial for clinical outcomes and quality of life. Many studies have shown promising results, including positive results for: psychotherapy, which included relaxation training, cognitive-behavioural stress management, and symptom control imagery training (Price et al., 1991; Zachariae et al., 1996), cognitive behavioural therapy (Fortune et al., 2002; Piaserico et al., 2016; Sijercic et al., 2020), hypnosis (Tausk and Whitmore, 1999), emotional disclosure (Vedhara et al., 2007; Paradisi et al., 2010), mindfulness based cognitive therapy (Fordham et al., 2015; Maddock et al., 2019), relaxation (Neerackal et al., 2020), and mindfulness meditation (Kabat-Zinn et al., 1998). A meta-analysis showed that overall, psychological interventions had a medium effect on the reduction in psoriasis severity (Lavda et al., 2012). Further evidence demonstrates that these interventions may be particularly effective for those with high levels of stress and more severe psychiatric diagnoses (Sijercic et al., 2020). Even if psychological interventions do not directly affect psoriasis severity, they can have substantial impacts on patients’ quality of life and psychological health (O'Leary et al., 2004; Zill et al., 2019).

Despite this promising evidence, many of these studies have important methodological limitations, such as small sample sizes, lack of comparator groups, and randomisation, no follow-ups, and high attrition rates and missing data (Lavda et al., 2012; Moon et al., 2013; Chen et al., 2014; Qureshi et al., 2019). In addition, most of these interventions are long-term (weeks to months) and therefore intensive for patients. Furthermore, clinical interventions usually require highly trained professionals. Few studies have investigated whether brief psychological interventions that do not rely on specialist delivery could also improve psoriasis.

One type of brief intervention that could be beneficial is the companion robot Paro. Paro is a robot designed to resemble a baby harp seal and provide comfort to users like a pet. Using sensors, Paro proactively and reactively responds to the user’s touch and voice through movements and noises (Wada et al., 2005). Paro also has a soft fur coat that provides tactile comfort when stroked. Interacting with Paro stimulates the user’s senses and evokes emotions through social bonding (Shibata and Coughlin, 2014). Patients with psoriasis have high rates of social anxiety and social avoidance due to stigmatisation (Schneider et al., 2013; Łakuta and Przybyła-Basista, 2017), and therefore it is hypothesised that Paro may be beneficial *via* this social bonding. Social interaction with Paro may be most beneficial for those patients with more severe and visible psoriasis, as these patients have higher rates of social anxiety (Kent and Keohane, 2001).

Research has shown that Paro can reduce perceived stress (McGlynn et al., 2016), stress hormone levels (Saito et al., 2003; Nomura and Hoshina, 2017), loneliness (Robinson et al., 2013), depressive symptoms (Bennett et al., 2017), pain (Geva et al., 2020), and blood pressure (Robinson et al., 2015), and improve mood (Inoue et al., 2013). Another study has found that interacting with the Paro robot for only 30 min can improve skin healing rates from an experimental tape-stripping wound after a stressor in people with healthy skin (Law et al., 2020a). This study indicated that there are direct benefits to skin healing by interacting with Paro. It was postulated that Paro may also be beneficial for patients with psoriasis.

The current study aimed to investigate whether interaction with Paro could decrease psychological stress and improve skin outcomes, measured *via* trans-epidermal water loss (TEWL) and Raman spectroscopy, after exposure to an experimental stressor in patients with psoriasis.

Trans-epidermal water loss indicates the ability of the skin to prevent water loss through the epidermis. A higher TEWL value indicates a higher amount of water is evaporating through the epidermis and a lower skin barrier function. TEWL is higher in psoriasis plaques compared to non-affected skin and rises with increased severity, indicating impaired barrier function (Rajka and Thune, 1976). Therefore, TEWL can provide a measure of psoriasis severity.

Raman spectroscopy measures the vibrational frequencies of molecules, and thus is sensitive to changes in the molecular composition of skin areas affected by psoriasis. In this technique, a small area of the skin surface is irradiated with a low power laser *via* a fibre optic probe. The light that is scattered inelastically by the biomolecules comprising the skin components is collected through the probe and analysed using a spectrometer. The measurement is non-invasive, non-painful, and rapid; each measurement takes 20 s. Raman spectroscopy has previously been investigated for distinguishing between benign and cancerous tissue in a variety of organs, including skin (Zhao et al., 2015), lung (Huang et al., 2003), larynx (Stone et al., 2000), prostate, gastric (Ouyang et al., 2015), colorectal (Li et al., 2014), and breast tissue (Vargas-Obieta et al., 2016).

It was hypothesised that after a stressor, all participants would have increased psychological stress levels and negative affect, and worsened skin outcomes including increased TEWL and increased levels of stress-induced biomarkers, compared with baseline levels. It was also hypothesised that those participants provided with Paro after the stressor will have improved positive affect and skin outcomes, with a reduction in the levels of stress-induced biomarkers in the psoriasis lesions, stress, and negative affect levels, compared to the control condition, which was not provided with the Paro robot.

## Materials and Methods

### Trial Registration

This study was registered at https://anzctr.org.au, identifier: ACTRN12619000681156.

### Design

A 3(time-point) × 2(condition) mixed factorial randomised trial was designed to assess the effects of Paro vs. a control condition on skin outcomes and psychological variables after a stressor. Recruitment occurred from August 2019 to June 2020.

### Sample

A sample of 25 adults with psoriasis (13 female, 12 male; average age = 43.00 years; range = 17–75 years) was recruited from the community using flyers, social media, and email advertisements. Participants were included if they were over the age of 16, were diagnosed with plaque psoriasis and spoke fluent English. Participants were excluded if they had any other chronic skin conditions, were taking any systemic therapy for their psoriasis (including cyclosporin, methotrexate, acitretin, or concurrent phototherapy or biologic agents), were taking any other immunosuppressive medications (e.g., systemic corticosteroids) or had any recent or anticipated changes in anti-depressant or anxiolytic medications.

Ethics approval was granted by the University of Auckland Human Participants Ethics Committee.

#### Power Analysis

The required sample size was calculated using the programme G*Power (Faul et al., 2007) for a 2(conditions) × 3(time-points) mixed ANOVA. A power level of 0.80 and alpha level of 0.05 were chosen. An expected effect size of *F* = 0.29 was estimated from Law et al. (2020a) (gave *N* = 64). The sample size was increased by 20% due to the possible errors in TEWL measurement, giving a total required sample size of 76. However, due to recruitment issues with the sample, alongside COVID-19 lockdown restrictions, recruitment for this study had to be ceased after only 25 participants. Therefore, this study can only be considered as a pilot study and proof of methodology, but is not powered to detect effects. The analysis of preliminary effects and the feasibility of the method are reported to inform further work.

### Procedure

**Figure 1:**
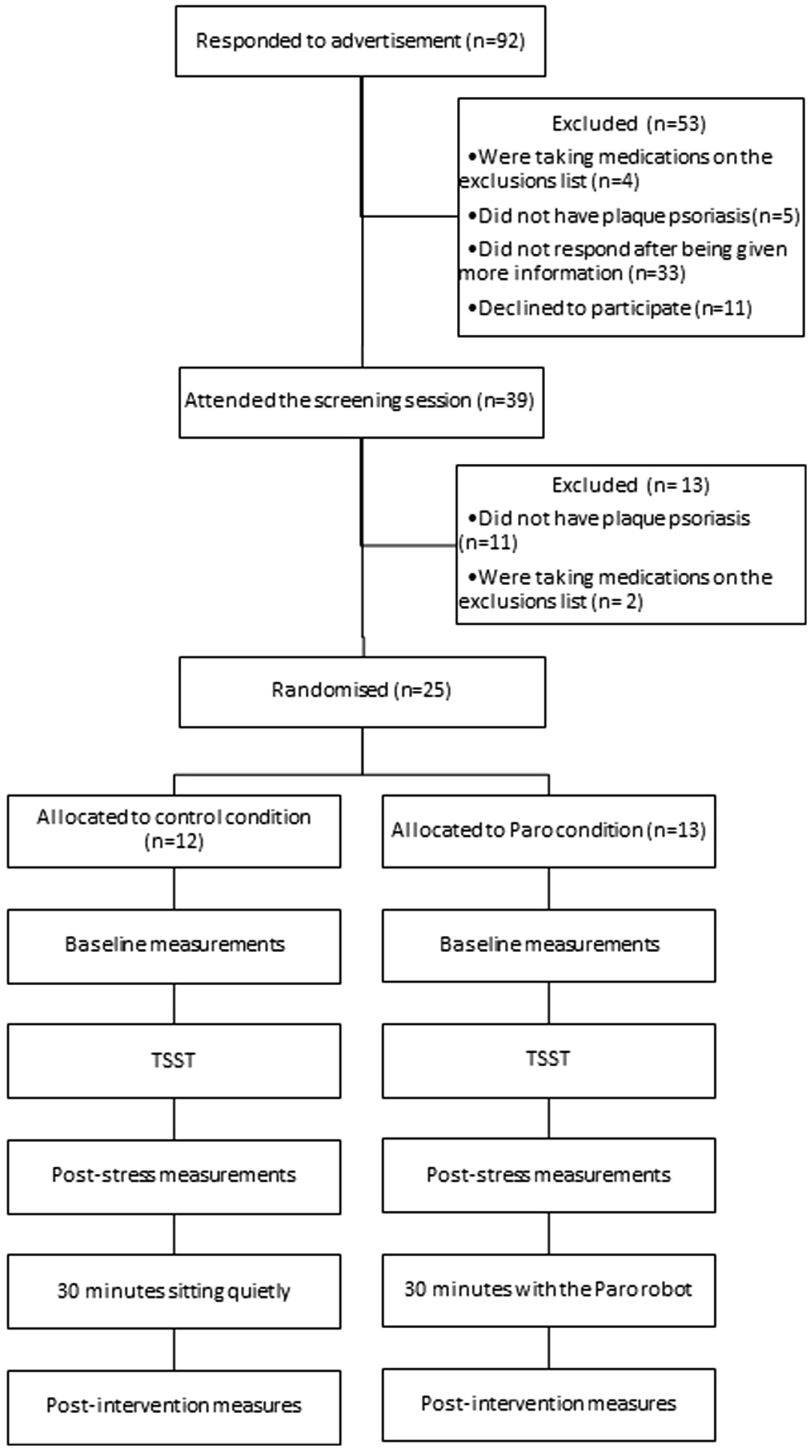
Overview of the study procedure.

The study procedure is provided in Figure 1. Interested participants first attended a 20-min screening session with a dermatologist to assess their eligibility, at the University of Auckland. After providing informed consent, the dermatologist assessed the participants’ skin to confirm the diagnosis of chronic plaque psoriasis and screened them for the other eligibility criteria. If the participant met all criteria, the dermatologist then completed the Psoriasis Area and Severity Index (PASI; Fredriksson and Pettersson, 1978), which is a validated and accepted measure of overall psoriasis severity. The PASI uses a formula to combine estimates of the percentage of area of skin involved, with scores for the three main clinical manifestations of psoriasis (erythema, induration, and desquamation) to get a score ranging from 0 to 72. Lastly, the dermatologist selected one psoriasis plaque to be used in the experimental session. The participants were asked to refrain from treating this selected plaque with any topical treatments or moisturisers until after the experimental session.

The second 90-min experimental session at the University of Auckland then occurred at least 1 week after the screening session. A dehumidifier was running in the room to minimise possible confounds in TEWL measurements due to high ambient humidity. To start the experimental session participants completed baseline measurements, including a questionnaire assessing demographics, health behaviours, questions about their psoriasis, and psychological measurements. Participants were then asked to lie on a bed and expose the selected plaque. The researcher marked out two 1 cm^2^ measurement areas; one on the selected plaque (the psoriasis site), and the other on uninvolved skin at least 1 cm away from the plaque (the control site). The researcher then measured each of these sites using the Tewameter to assess TEWL, and the Raman spectroscopy to assess the composition of the skin.

After these measurements, the participants were exposed to a shortened version of the Trier Social Stress Test (TSST) to elicit stress (Kirschbaum et al., 1993). This task has been shown to reliably induce both physiological and psychological stress in most people (Miller and Kirschbaum, 2013). Participants were given 3 min to prepare and 5 min to present an oral speech on why they deserve their dream job. They were told that their speech would be video-recorded and a panel of judges would review it and award the best speech with a $100 voucher. A shortened version of the TSST, with the inclusion of only the speech-task has been found to be just as effective in eliciting stress as the full version of the TSST (Goodman et al., 2017).

After the stressor, participants again completed measures of psychological variables, TEWL, and Raman spectroscopy before being randomised to one of two conditions (Paro or control). Randomisation was conducted by a researcher uninvolved in the study using a random number generator. Group allocation was concealed to the lead researcher up until this point, where the researcher opened a sealed envelope which contained the participants’ assigned condition. Participants were asked to interact with the Paro robot (Paro condition) or to sit quietly (control condition) during the 30-min intervention period, while they recovered from the stressor. Those in the Paro condition were asked to interact with the Paro robot in any way they wished over the intervention period. The Paro was turned on, and responded with different movements and sounds when the participant talked to it, stroked it, moved it, or touched its body, flippers, or whiskers. Participants in both groups were asked not to use any personal electronic devices, scratch the selected plaque or fall asleep during this period. The researcher left the room and returned once 30-min had elapsed. All measures were then completed for a final time and participants were provided with a $40 voucher at the completion of the session.

### Measures

#### Demographics and Health Behaviours

At baseline, participants were asked to report on their demographics, including gender, age, weight, height, and ethnicity. They were also asked to report on health behaviours, including alcohol consumption (indexed as on average, how many standard drinks they had per week), exercise levels (on average, how many days a week they engage in 30 min or more of physical activity), the quality of their diet over the past week from 1(very poor) to 5(very good), and on average, how many hours of sleep they had per night.

#### Psoriasis-Specific Questions

At baseline, participants were asked to answer questions about their psoriasis including the age they experienced their first outbreak and how many years they had psoriasis. They were also asked to state whether they believed that stress frequently worsens the severity of their psoriasis, with answers including yes, no, and unsure. Lastly, they were asked to put a cross on a 100 mm visual analogue scale to represent how severe they believed their psoriasis was right now. The anchors on the scale were no symptoms to worst symptoms imaginable.

#### Perceived Stress

The 10-item Perceived Stress Scale (PSS; Cohen et al., 1994) was used to evaluate the extent to which participants viewed their life as being stressful. Participants were asked how much they had felt a certain way over the last month on a scale of 0(never) to 4(very often). Scores were totalled to give a perceived stress score ranging from 0 to 40.

#### Depression

The Patient Health Questionnaire-9 (PHQ-9; Spitzer et al., 1999) was used to measure depression levels at baseline. Participants were asked over the last 2 weeks, how often they had been bothered by a set of problems congregated from the DSM-IV depression criteria on a scale of 0(not at all) to 3(nearly every day). Scores were totalled to give a depression score ranging from 0 to 27.

#### Affect

Affect levels were measured at baseline, after the stressor and after the intervention period using the Positive and Negative Affect Schedule (PANAS; Watson et al., 1988). This scale consists of two 10-item scales, measuring current positive and negative affect levels. Participants were asked to rate the extent they felt certain emotions right now, on a scale of 1(not at all) to 5(very much). The scores for each scale were totalled to give a score for both positive affect and negative affect ranging from 10 to 50.

#### Visual Analogue Scales

Self-reported stress, relaxation, and stimulation were measured at baseline, after the stressor and after the intervention period using 100 mm visual analogue scales. Participants were asked to place a cross on a 100 mm line to indicate how they were currently feeling. The anchors on the scales were not at all stressed to extremely stressed; not at all relaxed to very relaxed; and very bored to very stimulated. These visual analogue scales have been used successfully in previous research (Law et al., 2020a,b).

#### Trans-Epidermal Water Loss

Trans-epidermal water loss was measured at the two sites, at three time-points (baseline, after the stressor, and after the intervention period) using a Tewameter TM300 probe (Courage + Khazaka, Germany), which measures the evaporation rate (in g/m^2^h) in the air layer adjacent to the skin (Meesters et al., 2018). Before the measurements, the Tewameter probe was heated to 34°C by a probe heater to ensure it was at skin temperature. Once heated, the probe was placed against each of the two marked sites, measuring each site for 60 s, with one measurement being taken every second. Twenty consecutive measurements with a SD below 0.5 were averaged to give an overall TEWL value for both the psoriasis and control sites for each of the three time-points.

#### Raman Spectroscopy

Raman spectra were recorded *in vivo* from participants at baseline, after the stressor and after the intervention period. Spectra were recorded using an EmVision Raman fibre optic probe and EmVision spectrometer, equipped with 1,800 g/mm grating, a TEC CCD detector, 50 μm slit width, and using 830 nm excitation at 40 mW. Five repeat spectra were recorded for 10–20 s from a 1.2 mm area on the skin of the 25 participants in each of the three time-points, from both the psoriasis and control sites, resulting in 375 spectra in total.

### Statistical Analysis

The psychological and TEWL data were analysed using IBM SPSS Statistics 26. All analyses were conducted by original assigned groups. Mixed factorial ANOVAs were conducted to analyse the interaction and main effects of time-point and condition on the TEWL data and the psychological variables, including the visual analogue scales and the PANAS.

The Raman spectra were processed using an in-house Matlab algorithm to remove the fibre optic interference signal, and baseline corrected using a penalised asymmetric least squares algorithm (Eilers, 2003), after which the spectra were normalised to the peak position at 1,436 cm^−1^. Principal component analysis (PCA) and ANOVAs were performed using MATAB and PLS Toolbox 8.1 (EigenVector technologies).

## Results

### Baseline Characteristics

The baseline and demographic characteristics for the sample are shown in Table 1; no significant differences were found between conditions.

**Table 1:** Summary of demographic and baseline characteristics across conditions.

Baseline variable	Control (*n* = 12)	Paro (*n* = 13)	Total (*n* = 25)	Value of *p*
Age (years), *M*(*SD*)	41.25(18.66)	44.62(19.62)	43.00(18.84)	0.665^a^
Sex, *n*(%)				
Female	7(58%)	6(46%)	13(52%)	0.543^b^
Male	5(42%)	7(54%)	12(48%)	
BMI, *M*(*SD*)	24.24(5.01)	27.29(5.79)	25.83(5.53)	0.193^a^
Exercise (days/week), *M*(*SD*)	4.58(2.11)	3.23(2.45)	3.88(2.35)	0.155^a^
Diet Quality, *M*(*SD*)	3.67(0.65)	3.46(0.78)	3.56(0.71)	0.483^a^
Sleep (hours/night), *M*(*SD*)	7.11(1.05)	6.50(1.14)	6.79(1.12)	0.179^a^
PSS, *M*(*SD*)	12.67(6.91)	13.15(4.18)	12.92(5.54)	0.831^a^
PHQ-9, *M*(*SD*)	12.75(2.14)	13.54(2.90)	13.16(2.54)	0.451^a^
Selected Psoriasis Location, *n*(%)				
Leg	5(42%)	1(8%)	6(24%)	0.067^b^
Arm	2(16%)	7(54%)	9(36%)	
Torso	5(42%)	5(38%)	10(40%)	
PASI Score, *M*(*SD*)	7.26(5.70)	6.45(6.39)	6.84(5.95)	0.743^a^
Self-reported Psoriasis Severity, *M*(*SD*)	36.33(22.54)	44.62(25.92)	40.64(24.22)	0.405^a^
Age of Psoriasis Onset, *n*(%)				
0–13	3(25%)	3(23%)	6(24%)	0.903^b^
14–19	3(25%)	5(38%)	8(32%)	
20–29	5(42%)	4(31%)	9(36%)	
Over 30	1(8%)	1(8%)	2(8%)	
Years with Psoriasis, *n*(%)				0.257^b^
<5 years	2(17%)	0(0%)	2(8%)	
5–10 years	3(25%)	2(15%)	5(20%)	
10–20 years	1(8%)	4(31%)	5(20%)	
<20 years	6(50%)	7(54%)	13(65%)	

*M*, mean, *SD*, standard deviation, %, and percentage of participants in that category. *p* value was calculated by independent samples *T*-Tests^a^ and Chi-square tests.^b^

As shown in Table 1, on average, the sample had a dermatologist rated PASI of 6.84 which represents moderate psoriasis. However, this score ranged from 1.20 to 21.30, indicating a large range in psoriasis severity across the sample. Self-reported psoriasis severity, reported *via* a 100-point visual analogue scale, followed a similar pattern with a mean rating of 40.64 (range = 6.00–80.00). The dermatologist and self-reported psoriasis severity ratings were significantly correlated with a large effect size (*r* = 0.70, *p* < 0.001).

On average, the sample had moderate depression, based on known cut-off scores for the PHQ-9 (Spitzer et al., 1999). The average depression score of the sample (*M* = 13.16) was higher than average depression levels in the general population (*M* = 2.91; Kocalevent et al., 2013), in concurrence with the literature on depression levels for patients with psoriasis. However, the sample had an average perceived stress score (*M* = 12.92) similar to the general population (*M* = 14.66; Cohen and Janicki-Deverts, 2012), indicating that this sample was not particularly stressed.

### Trans-Epidermal Water Loss

A paired samples *T*-test was conducted to examine whether there was a difference in TEWL between the psoriasis and control sites at baseline. As expected, the psoriasis site had significantly higher baseline TEWL (*M* = 31.70, *SD* = 9.81) than the control site [*M* = 17.58, *SD* = 4.41, *t*_(23)_ = 6.85, *p* < 0.001], indicating an impaired skin barrier function in psoriatic plaques compared to non-affected skin.

Mixed ANOVAs were conducted for both the psoriasis and control sites to evaluate the effects of time-point and condition on TEWL. There were no significant main effects of time-point [control site; *F*_(2,35)_ = 1.70, *p* = 0.201, *η_p_^2^* = 0.07, psoriasis site; *F_(2,44)_* = 1.86, *p* = 0.168, *η_p_^2^* = 0.08] or condition [control site; *F_(2,35)_* = 1.03, *p* = 0.351, *η_p_^2^* = 0.04, psoriasis site; *F_(1,22)_* = 0.21, *p* = 0.650, *η_p_^2^* = 0.01] for either site. However, the medium effect sizes for time-point for both sites suggest that with a larger sample size, these effects could become significant.

There were also no significant interaction effects for condition × time-point for either the control [*F_(1,22)_* = 1.40, *p* = 0.249, *η_p_^2^* = 0.06] or psoriasis sites [*F_(2,44)_* = 0.28, *p* = 0.753, *η_p_^2^* = 0.01]. The summary statistics for these tests are provided in Tables 2, 3. The medium effect size for the control site indicates that this interaction effect could be significant in a larger sample. As shown in Table 3, this is trending in favour of lower TEWL for the Paro condition at the post-intervention time-point.

**Table 2:** Summary statistics and *post-hoc* comparisons for the outcomes across time, irrespective of condition.

Task		Baseline	Post-stressor	Post-intervention
TEWL	Control site, *M*(*SD*)	17.58(4.41)	18.60(4.91)	17.65(3.62)
	Psoriasis site, *M*(*SD*)	31.70(9.81)	32.40(9.00)	30.71(9.49)
Visual analogue scales	Stress, *M*(*SD*)	26.92(22.67)b^*^c^*^	41.44(27.42)a^*^c^**^	11.60(13.68)a^*^b^**^
	Relaxation, *M*(*SD*)	75.32(15.36)b^*^c^*^	52.92(28.94)a^*^c^**^	83.72(17.68)a^*^b^**^
	Stimulation, *M*(*SD*)	65.20(20.52)b^*^c^*^	77.68(16.01)a^*^c^**^	40.48(27.96)a^*^b^**^
PANAS	Positive affect, *M*(*SD*)	31.40(8.56)c^*^	32.40(8.54)c^**^	25.52(9.68)a^*^b^**^
	Negative affect, *M*(*SD*)	13.44(5.44)b^*^c^**^	16.16(6.11)a^*^c^**^	11.32(3.79)a^**^b^**^

a, different to baseline, b, different to post-stressor, and c, different to post-intervention.

* *p* < 0.05;

** *p* < 0.001.

*M*, mean, *SD*, standard deviation, and TEWL, trans-epidermal water loss.

**Table 3:** Summary statistics for the outcomes at the post-intervention time-point across condition.

Condition		Paro	Control
TEWL	Control site, *M*(*SD*)	17.21(3.34)	18.09(3.97)
	Psoriasis site, *M*(*SD*)	30.20(10.86)	31.21(8.36)
Visual analogue scales	Stress, *M*(*SD*)	15.38(16.53)	7.50(8.66)
	Relaxation, *M*(*SD*)	77.31(20.32)	90.67(11.40)
	Stimulation, *M*(*SD*)	42.54(29.41)	38.25(27.42)
PANAS	Positive affect, *M*(*SD*)	25.15(8.44)	25.92(11.24)
	Negative affect, *M*(*SD*)	12.00(5.20)	10.58(0.90)

*M*, mean, *SD*, standard deviation, and TEWL, trans-epidermal water loss, there were no significant differences in any outcomes between conditions (all *ps* > 0.5).

Bayesian independent samples normal tests were conducted at the post-intervention time-point to further test this hypothesis. The resulting Bayes factors at both the control (*B* = 3.02) and psoriasis sites (*B* = 3.39) were both above in the section “Baseline Characteristics,” and therefore support the alternative hypothesis by providing evidence that the control and Paro conditions had different TEWL levels at the post-intervention time-point.

### Visual Analogue Scales

Mixed ANOVAs showed main effects of time-point for all psychological variables; stress [*F_(2,40)_* = 14.92, *p* < 0.001, *η_p_^2^* = 0.39], relaxation [*F_(1,21)_* = 22.72, *p* < 0.001, *η_p_^2^* = 0.50], and stimulation [*F_(1,21)_* = 60.44, *p* < 0.001, *η_p_^2^* = 0.48], with large effect sizes. The *post-hoc* tests are provided in Table 2. Irrespective of condition, the stressor led to an increase in stress and stimulation levels, and a decrease in relaxation. The intervention period resulted in a decrease in stress and stimulation to below baseline levels and an increase in relaxation to above baseline levels. There were no significant main effects for condition or interaction effects for any of the psychological variables. The means across condition at the post-intervention time-point are shown in Table 3.

### Positive and Negative Affect Schedule

Mixed ANOVAS showed main effects for time-point for both positive [*F_(1,31)_* = 15.12, *p* < 0.001, *η_p_^2^* = 0.40] and negative affect [*F_(2,35)_* = 26.46, *p* < 0.001, *η_p_^2^* = 0.53], with large effect sizes. The *post-hoc* tests are provided in Table 2. Irrespective of condition, the stressor led to an increase in negative affect, and the intervention led to a decrease in both positive and negative affect to below baseline levels. There were no significant main effects for condition or interaction effects. The means across condition for the post-intervention time-point are shown in Table 3.

### Raman Spectroscopy

**Figure 2:**
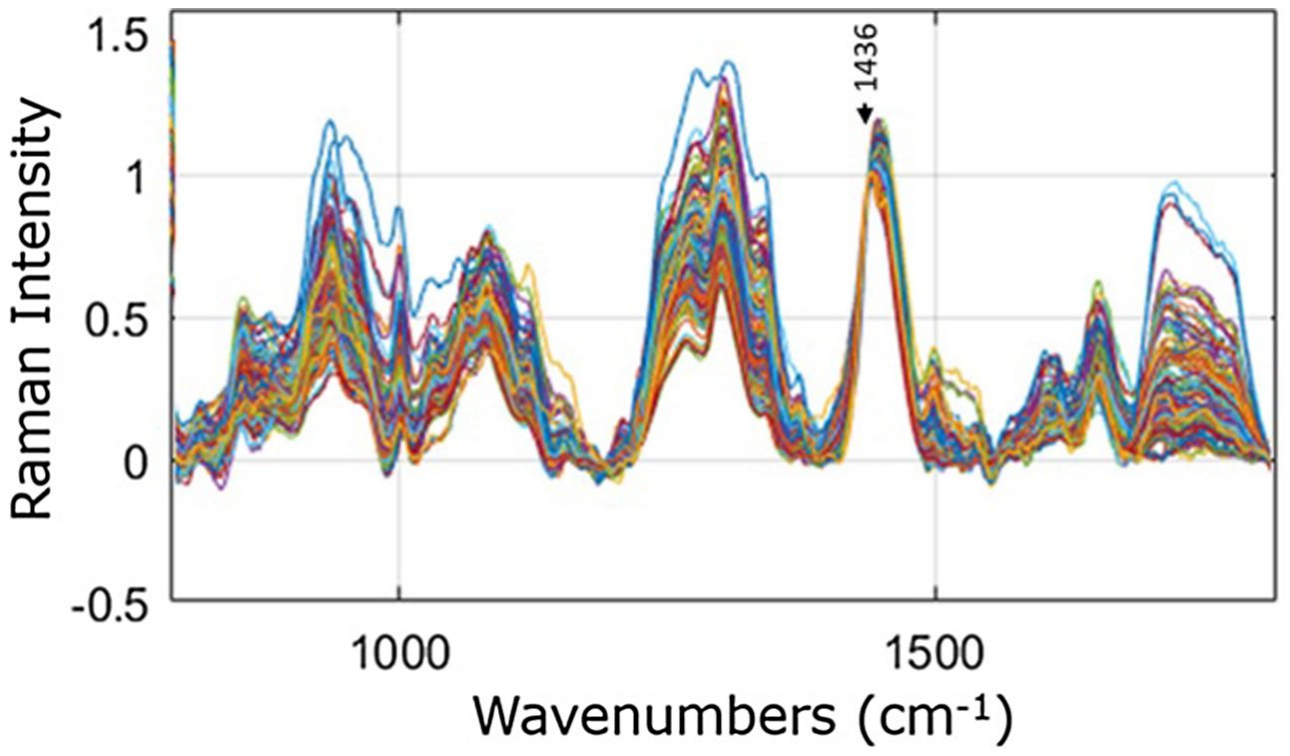
Raman spectra recorded of psoriasis and control sites from all 25 participants, overlaid for comparison and normalised to the peak height at 1,436 cm^−1^, indicated by the arrow.

The 375 spectra recorded of the psoriasis and control sites from the 25 participants are shown in Figure 2, after removal of the fibre optic interference signal, baseline correction, and normalization to the peak position at 1,436 cm^−1^. Large variations in the relative peak intensities of the spectral peaks are evident. These variations are due mainly to differences in skin types, the different body areas analysed, and the compositional differences between the psoriasis and control sites.

#### Analysis of the Raman Spectra

The Raman spectra were analysed to investigate whether any changes in the molecular vibrations of the psoriasis regions had occurred over the three time-points. The five repeat spectra recorded at each time-point and site were averaged, and then the control site was subtracted from the psoriasis site, resulting in 75 difference spectra: three difference spectra for the three time-points, for each of the 25 participants.

**Figure 3:**
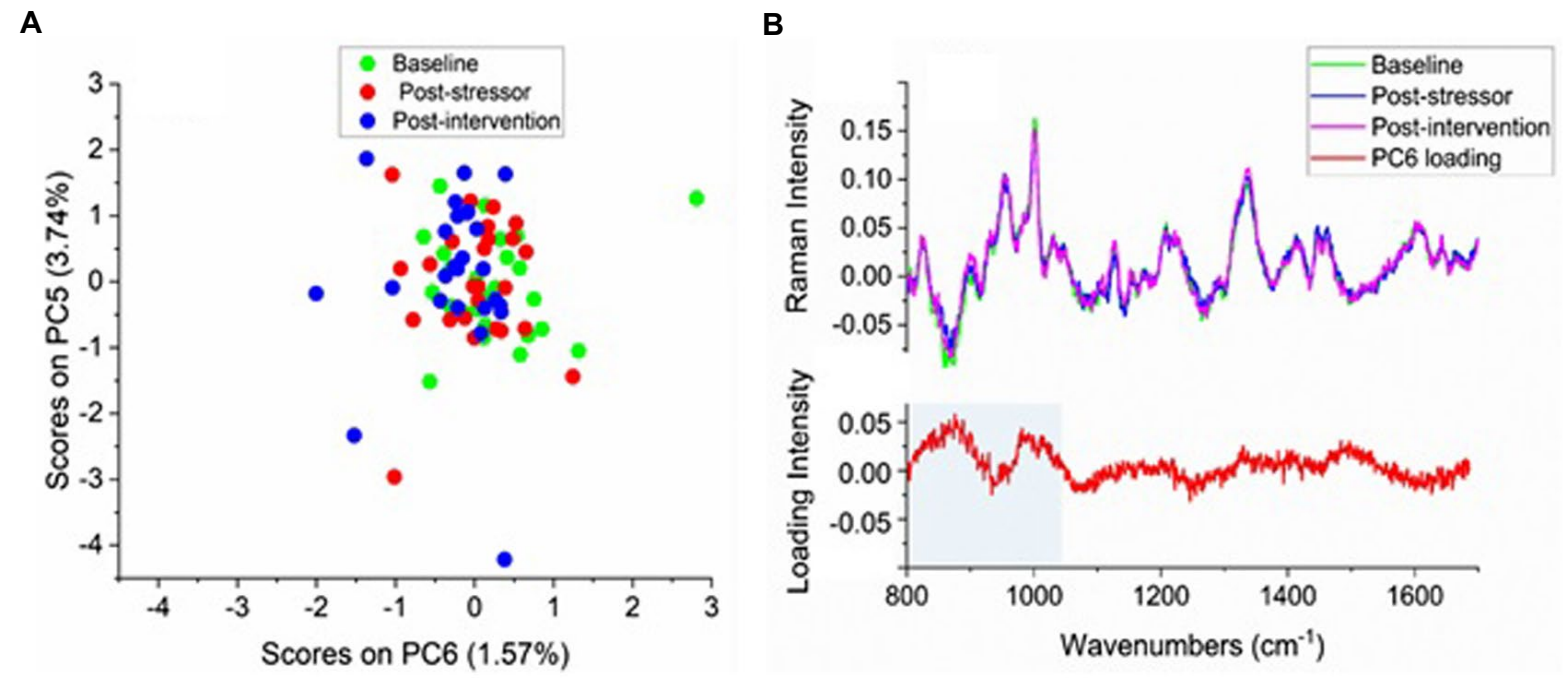
**(A)** Principal component analysis (PCA) scores colour coded according to time session and **(B)** Top: the averaged difference spectra (averaged psoriasis—averaged control) for each time-point (light green: baseline, blue: post-stressor, pink: post-intervention), **(B)** Bottom: loading plot for PC6, with shaded region corresponding to positive loadings for this PC.

A PCA of the 75 difference spectra between the psoriasis and control sites was performed (after mean-centering of the difference spectra to explore similarities or differences between the three time-points). About 95% of the variance in the spectra was described by 12 principal components (PC’s). No clear clustering of the sample scores according to the three time-points was evident. However, as a group most of the post-intervention time-point samples (blue) appear slightly more toward the left-hand side of the *X* axis in Figure 3A, which indicates they have lower values for the positive loadings of the sixth principal component (PC6). The adjacent loading plot in Figure 3B (bottom) indicates, for each wavenumber, the extent to which the scores are associated with that PC. The positive “peaks” in the loading plot for PC6 (red curve) represent those wavenumbers, and hence molecular species, that are affiliated more with the red post-stressor and green baseline time-point samples, while the blue post-intervention time-point sample scores appear on the negative side of PC6 in the PCA score plot (Figure 3A), with the higher loadings corresponding with wavenumbers between 820 and 1,000 cm^−1^ (Figure 3B).

While the grouping of post-intervention scores on the negative side of PC6 accounts for only 5.3% of the total variance and around one third of the scores from each of baseline and post-stressor time-points also show higher negative loadings for these compounds, PC6 was the highest PC that showed grouping for any of the time-points (with PC1 being the highest loading overall). The scores for PCs 1–5 showed no grouping for the three time-points; the PC1 loadings represented the mean, accounting for differences in overall spectral intensities between some of the individual participants from the others, while PC loadings 2–4 showed differences mainly in the 1,450 cm^−1^ lipid region, amide I and III bands and phenylalanine band at 1,003 cm^−1^, also for a few individuals only as indicated by the scores plots.

To test how significant the increased intensities in the difference spectra of the post-intervention time-point were compared to the baseline and post-stressor time-points, an ANOVA was performed on the band areas in the region 820–980 cm^−1^ which displayed the highest negative loadings (marked in Figure 3B). There were no significant differences between any of the time-points; baseline and post-stressor (*p* = 0.977, *d* = 0.38), baseline and post-intervention (*p* = 0.127, *d* = 0.78), and post-stressor and post-intervention (*p* = 0.143, *d* = 0.47).

A two-way ANOVA (time-point vs. condition) of four main peak areas in the difference spectra was also performed on each of the areas of the peaks at 1,340, 1,001, 958, and 901 cm^−1^ (labelled in the difference spectra shown in the lower plot Figure 3B) for the 25 difference spectra. No significant effects were found in these peak areas or peak area ratios, on any of these peak areas with time-points or between the Paro and control conditions.

## Discussion

This randomised pilot study investigated the feasibility of interacting with a Paro robot after a stressor on skin outcomes and psychological variables in patients with psoriasis. Although the study was planned as a full randomised control trial, recruitment issues meant the study was completed as a pilot, and therefore meaningful conclusions about the effects of a Paro robot on psoriasis cannot be drawn from this study. However, feasibility and effect sizes are reported to inform future research.

Significant effects of the experimental manipulations were seen, demonstrating feasibility of the experimental paradigm. The stress task significantly increased stress, stimulation, and negative affect levels and decreased relaxation, in both conditions, as expected. The intervention period, on the other hand, led to a decrease in stress, stimulation, and negative affect and positive affect to below baseline levels, and an increase in relaxation to above baseline levels. This indicates that the method worked to induce stress and promote stress recovery.

Trans-epidermal water loss was significantly different between the psoriasis and control sites, which was expected, and indicates that TEWL is a valid measure of psoriasis. There were no significant differences in TEWL across the experimental session or between conditions. However, effect sizes and Bayesian statistics suggest that these effects could become significant in a fully powered sample, suggesting feasibility of this measure in larger trials. The Raman results indicated a trend towards differences across the time-points; however, no differences across conditions were found.

Although previous research has suggested that Paro can decrease stress (Saito et al., 2003; McGlynn et al., 2016; Nomura and Hoshina, 2017) and improve skin outcomes (Law et al., 2020a), no significant effects of condition were seen in this pilot study. This lack of effects could be due to a variety of reasons. Most importantly, the study was not powered to detect any intervention effects. The effect size for the TEWL measurements between conditions in the non-psoriatic skin suggests that a significant effect may be detected in a larger sample of at least 114 participants.

As well as the low sample size, there was large individual variability in the sample. Research has demonstrated that psychological interventions may be more beneficial for certain patients. For example, a recent meta-analysis demonstrated that effect sizes from cognitive-behavioural interventions for patients with psoriasis were highest in those patients with higher psychopathology pre-treatment (Sijercic et al., 2020). The current sample had a wide range of depression and stress scores. Therefore, it may be that Paro is only effective for those with higher depression and stress. Paro may be less effective for those who are already psychologically adaptive. The meta-analysis also found that effect sizes were larger for those with more severe psoriasis (Sijercic et al., 2020). The patients in the current study had PASI scores ranging from very mild to very severe; however, many only had mild psoriasis, as is common in the wider community. Psoriasis may be less psychologically damaging to those with lower PASI scores, especially if it can easily be hidden. Therefore, the psychological and stress-reducing effects of Paro may be more effective in those with more severe psoriasis.

Paro was hypothesised to have an effect on healing due to the beneficial effects of social interaction with the robot. However, research has shown that patients with psoriasis may not benefit as much physiologically from social inclusion interventions, compared to controls (Ponsi et al., 2019), despite the high rates of social anxiety and avoidance in these patients. Therefore, other forms of psychological interventions may be more beneficial in these patients. Lastly, the literature demonstrates that patients with psoriasis have differences in stress-reactivity (Zachariae et al., 2004). Although stress may trigger flare-ups in stress-responders, this may not be seen in non-stress responders, and therefore psychological interventions to reduce stress may not be effective in these patients (Fordham et al., 2013). The current study did ask participants to report whether they believed stress worsened their psoriasis; however, there were only 16 stress-responders and one non stress-responder, with the rest of the sample reporting that they were unsure. Therefore, the recruitment of only stress-responders may lead to stronger effects. Despite these propositions about individual variability effects, the sample size for this study was too small to complete any sub-analyses on these possible individual factors. Future research should ensure a large enough sample to be able to examine these possibilities.

Another possible explanation for the null results of condition could be the brief time-frame of the intervention. A one-off 30-min intervention may not be long enough to see significant biological changes in the skin that could be measured by Raman spectroscopy, especially for psoriasis, which is a chronic and long-term disease. The physiological stress response is an evolutionarily mechanism which induces a rapid response in the face of a threat. Therefore, an acute change in physiology would be expected, but may not have been significantly detected by the Raman technique. Previous research on psychological interventions for psoriasis tends to use longer interventions ranging from weeks to months. This study attempted to investigate whether a short-term intervention could have a similar benefit; however, the lack of effects may indicate that this is not possible. Raman spectroscopy has also not been previously used in short-term studies to investigate acute changes in the skin from psychological factors. Future research could therefore investigate the effects of Paro on psoriasis outcomes over a long-term study in order to detect any significant changes in psoriasis severity over time.

Despite the lack of significant effects between conditions, this study did demonstrate significant changes in the outcomes over the experiment, therefore indicating the feasibility of the experimental paradigm for future research. Even with a limited sample size, this study showed that the stress task was successful at changing the participants’ mood and stress levels and in contrast, the intervention period allowed for successful stress-reduction to below basal levels. Therefore, this paradigm is successful at changing short-term psychological outcomes in patients with psoriasis. However, stress induction and recovery were observed in both conditions and therefore, the TSST may have been too stressful to observe any significant intervention effects. Future research may investigate this intervention in patients experiencing more naturalistic stressors.

The Raman analysis was also seen to be feasible. The lower PCA scores as a group of the post-intervention time-point difference spectra for the positive loadings for PC6 suggest that the psoriasis sites after the intervention showed differences in the vibrational energies of the biomolecules from the baseline and post-stressor time-points. This band region includes the C-C ring stretching vibrations of a number of amino acids, which in skin would be primarily proline, hydroxyproline (Erckens et al., 1997;Talari et al., 2015; Hernández et al., 2016), tyrosine, and tryptophan (Talari et al., 2015; Hernández et al., 2016). The latter two compounds are among the cytokines which are increased in inflammatory skin conditions (Portugal-Cohen et al., 2012; de la O-Cuevas et al., 2019). Although none of the effects of time-point on the loading areas in this region were significant, the value of *p*s close to 0.1 between baseline and post-intervention, and between post-stressor and post-intervention support the preference for this region of post-intervention scores. The medium to large effect size of PC6 score between the baseline and post-intervention time-points shows that the intervention period, in both conditions, reduced proteins associated with cytokines that are increased in inflammatory skin conditions such as psoriasis, from baseline level. This is an important finding as reducing these pro-inflammatory cytokines can lead to a reduction in psoriasis severity and symptoms.

The strict eligibility criteria for the study led to low recruitment and the exclusion of many patients with psoriasis. For example, the study protocol excluded participants on any form of systemic therapy or immunosuppressive medication for psoriasis to minimise a confounding effect. These criteria were made clear on any advertisements and therefore, these patients may not have approached the researchers. This may have limited the sample of the study to only participants with milder psoriasis, as those with severe psoriasis are more likely to be on systemic treatment. Future research should include these patients as well to further expand the sample and improve recruitment rates.

In conclusion, this pilot study did not significantly demonstrate that interacting with a Paro robot after a stressor could influence skin outcomes or psychological variables in patients with psoriasis compared to a control condition, who did not receive a Paro robot. However, effect sizes for both the TEWL and Raman measurements suggest feasibility for a larger study that may be able to find significant effects across conditions. This pilot study also indicates the feasibility of the experimental methodology, as expected significant findings with medium effect sizes were observed from the experimental manipulations. Future research should investigate this work in larger samples of people who are experiencing significant stress, with sub-group analyses using a longer-term intervention period. Even if the Paro robot is not found to directly affect psoriasis severity, it could have substantial impacts on patients’ quality of life and psychological health, which should be investigated further.

## Data Availability Statement

The raw data supporting the conclusions of this article will be made available by the authors, without undue reservation.

## Ethics Statement

The studies involving human participants were reviewed and approved by University of Auckland Human Participants Ethics Committee. The patients/participants provided their written informed consent to participate in this study.

## Author Contributions

ML: conceptualization, data curation, formal analysis, investigation, methodology, project administration, visualization, and writing—original draft. PJ: conceptualization, investigation, methodology, supervision, and writing—review and editing. MN: conceptualization, data curation, formal analysis, investigation, resources, and writing—original draft. HH: conceptualization, investigation, and writing—review and editing. CG: formal analysis, investigation, and writing—review and editing. EB: conceptualization, methodology, resources, supervision, and writing—review and editing. All authors contributed to the article and approved the submitted version.

## Conflict of Interest

The authors declare that the research was conducted in the absence of any commercial or financial relationships that could be construed as a potential conflict of interest.

## Publisher’s Note

All claims expressed in this article are solely those of the authors and do not necessarily represent those of their affiliated organizations, or those of the publisher, the editors and the reviewers. Any product that may be evaluated in this article, or claim that may be made by its manufacturer, is not guaranteed or endorsed by the publisher.

## References

[ref1] Albanesi C. Madonna S. Gisondi P. Girolomoni G. (2018). The interplay between keratinocytes and immune cells in the pathogenesis of psoriasis. Front. Immunol. 9:1549. doi: 10.3389/fimmu.2018.01549, PMID: 30034395 PMC6043636

[ref2] Bennett C.C. Sabanovic S. Piatt J.A. Nagata S. Eldridge L. Randall N. (2017). “A robot a day keeps the blues away.” in *2017 IEEE International Conference on Healthcare Informatics (ICHI) August 2017*, IEEE; 536–540.

[ref3] Bundy C. Pinder B. Bucci S. Reeves D. Griffiths C. E. Tarrier N. (2013). A novel, web-based, psychological intervention for people with psoriasis: the electronic targeted intervention for psoriasis (eTIPs) study. Br. J. Dermatol. 169, 329–336. doi: 10.1111/bjd.12350, PMID: 23551271

[ref4] Chen Y. Xin T. Cheng A. S. (2014). Evaluating the effectiveness of psychological and/or educational interventions in psoriasis: a narrative review. J. Dermatol. 41, 775–778. doi: 10.1111/1346-8138.12583 25109476

[ref5] Cohen S. Janicki-Deverts D. (2012). Who’s stressed? Distributions of psychological stress in the United States in probability samples from 1983, 2006, and 2009. J. Appl. Soc. Psychol. 42, 1320–1334. doi: 10.1111/j.1559-1816.2012.00900.x

[ref6] Cohen S. Kamarck T. Mermelstein R. (1994). “Perceived stress scale,” in Measuring Stress: A Guide for Health and Social Scientists, *Vol*. 10, 1–2. Oxford University Press.

[ref7] de la O-Cuevas E. Badillo-Ramírez I. Islas S. R. Araujo-Andrade C. Saniger J. M. (2019). Sensitive Raman detection of human recombinant interleukin-6 mediated by DCDR/GERS hybrid platforms. RSC Adv. 9, 12269–12275. doi: 10.1039/C9RA01396B 35515877 PMC9063685

[ref8] Eilers P. H. (2003). A perfect smoother. Anal. Chem. 75, 3631–3636. doi: 10.1021/ac034173t, PMID: 14570219

[ref9] Erckens R. J. Motamedi M. March W. F. Wicksted J. P. (1997). Raman spectroscopy for non-invasive characterization of ocular tissue: potential for detection of biological molecules. J. Raman Spectrosc. 28, 293–299. doi: 10.1002/(SICI)1097-4555(199705)28:5<293::AID-JRS47>3.0.CO;2-0

[ref10] Faul F. Erdfelder E. Lang A. G. Buchner A. (2007). G* power 3: a flexible statistical power analysis program for the social, behavioral, and biomedical sciences. Behav. Res. Methods 39, 175–191. doi: 10.3758/BF03193146, PMID: 17695343

[ref11] Fordham B. Griffiths C. E. Bundy C. (2013). Can stress reduction interventions improve psoriasis? A review. Psychol. Health Med. 18, 501–514. doi: 10.1080/13548506.2012.736625, PMID: 23116223

[ref12] Fordham B. Griffiths C. E. Bundy C. (2015). A pilot study examining mindfulness-based cognitive therapy in psoriasis. Psychol. Health Med. 20, 121–127. doi: 10.1080/13548506.2014.902483, PMID: 24684520

[ref13] Fortune D. G. Richards H. L. Kirby B. Bowcock S. Main C. J. Griffiths C. E. (2002). A cognitive-behavioural symptom management programme as an adjunct in psoriasis therapy. Br. J. Dermatol. 146, 458–465. doi: 10.1046/j.1365-2133.2002.04622.x, PMID: 11952546

[ref14] Fortune D. G. Richards H. L. Kirby B. McElhone K. Markham T. Rogers S.. (2003). Psychological distress impairs clearance of psoriasis in patients treated with photochemotherapy. Arch. Dermatol. 139, 752–756. doi: 10.1001/archderm.139.6.752, PMID: 12810506

[ref15] Fortune D. G. Richards H. L. Main C. J. Griffiths C. E. (2000). Pathological worrying, illness perceptions and disease severity in patients with psoriasis. Br. J. Health Psychol. 5, 71–82. doi: 10.1348/135910700168775

[ref16] Fredriksson T. Pettersson U. (1978). Severe psoriasis–oral therapy with a new retinoid. Dermatology 157, 238–244. doi: 10.1159/000250839 357213

[ref17] Geva N. Uzefovsky F. Levy-Tzedek S. (2020). Touching the social robot PARO reduces pain perception and salivary oxytocin levels. Sci. Rep. 10, 9814–9815. doi: 10.1038/s41598-020-66982-y, PMID: 32555432 PMC7299999

[ref18] Gisondi P. Bellinato F. Girolomoni G. (2020). Topographic differential diagnosis of chronic plaque psoriasis: challenges and tricks. J. Clin. Med. 9:3594. doi: 10.3390/jcm9113594, PMID: 33171581 PMC7695211

[ref19] Goodman W. K. Janson J. Wolf J. M. (2017). Meta-analytical assessment of the effects of protocol variations on cortisol responses to the Trier social stress test. Psychoneuroendocrinology 80, 26–35. doi: 10.1016/j.psyneuen.2017.02.030, PMID: 28292684

[ref20] Heller M. M. Lee E. S. Koo J. Y. (2011). Stress as an influencing factor in psoriasis. Skin Therapy Lett. 16, 1–4. 21611682

[ref21] Hernández B. Coïc Y. M. Pflüger F. Kruglik S. G. Ghomi M. (2016). All characteristic Raman markers of tyrosine and tyrosinate originate from phenol ring fundamental vibrations. J. Raman Spectrosc. 47, 210–220. doi: 10.1002/jrs.4776

[ref22] Huang Z. McWilliams A. Lui H. McLean D. I. Lam S. Zeng H. (2003). Near-infrared Raman spectroscopy for optical diagnosis of lung cancer. Int. J. Cancer 107, 1047–1052. doi: 10.1002/ijc.11500, PMID: 14601068

[ref23] Inoue K. Nakamura M. Sakuma N. Okada M. (2013). Turning off or turning on?: two different ways to use a baby seal shaped robot Paro in occupational therapy for patients with dementia. Assist. Technol. Res. Ser. 33, 875–879. doi: 10.3233/978-1-61499-304-9-875

[ref24] Kabat-Zinn J. Wheeler E. Light T. Skillings A. Scharf M. J. Cropley T. G.. (1998). Influence of a mindfulness meditation-based stress reduction intervention on rates of skin clearing in patients with moderate to severe psoriasis undergoing photo therapy (UVB) and photochemotherapy (PUVA). Psychosom. Med. 60, 625–632. doi: 10.1097/00006842-199809000-00020, PMID: 9773769

[ref25] Kent G. Keohane S. (2001). Social anxiety and disfigurement: the moderating effects of fear of negative evaluation and past experience. Br. J. Clin. Psychol. 40, 23–34. doi: 10.1348/014466501163454, PMID: 11317946

[ref26] Kirschbaum C. Pirke K. M. Hellhammer D. H. (1993). The ‘Trier social stress test’–a tool for investigating psychobiological stress responses in a laboratory setting. Neuropsychobiology 28, 76–81. doi: 10.1159/000119004 8255414

[ref27] Kocalevent R. D. Hinz A. Brähler E. (2013). Standardization of the depression screener patient health questionnaire (PHQ-9) in the general population. Gen. Hosp. Psychiatry 35, 551–555. doi: 10.1016/j.genhosppsych.2013.04.006, PMID: 23664569

[ref28] Kotrulja L. Tadinac M. JokIć-BegIć N. Gregurek R. (2010). A multivariate analysis of clinical severity, psychological distress and psychopathological traits in psoriatic patients. Acta Derm. Venereol. 90, 251–256. doi: 10.2340/00015555-0838, PMID: 20526541

[ref29] Łakuta P. Przybyła-Basista H. (2017). Toward a better understanding of social anxiety and depression in psoriasis patients: The role of determinants, mediators, and moderators. J. Psychosom. Res. 94, 32–38. doi: 10.1016/j.jpsychores.2017.01.007, PMID: 28183400

[ref30] Lavda A. C. Webb T. L. Thompson A. R. (2012). A meta-analysis of the effectiveness of psychological interventions for adults with skin conditions. Br. J. Dermatol. 167, 970–979. doi: 10.1111/j.1365-2133.2012.11183.x, PMID: 22924999

[ref31] Law M. Jarrett P. Nater U. M. Skoluda N. Broadbent E. (2020a). The effects of sensory enrichment after a laboratory stressor on human skin barrier recovery in a randomized trial. Psychosom. Med. 82, 877–886. doi: 10.1097/PSY.0000000000000858, PMID: 32881761

[ref32] Law M. Jarrett P. Nater U. M. Skoluda N. Broadbent E. (2020b). The effects of environmental enrichment on skin barrier recovery in humans: a randomised trial. Sci. Rep. 10:9829. doi: 10.1038/s41598-020-66687-2, PMID: 32555211 PMC7299948

[ref33] Li S. Chen G. Zhang Y. Guo Z. Liu Z. Xu J.. (2014). Identification and characterization of colorectal cancer using Raman spectroscopy and feature selection techniques. Opt. Express 22, 25895–25908. doi: 10.1364/OE.22.025895, PMID: 25401621

[ref34] Maddock A. Hevey D. D’Alton P. Kirby B. (2019). A randomized trial of mindfulness-based cognitive therapy with psoriasis patients. Mindfulness 10, 2606–2619. doi: 10.1007/s12671-019-01242-3

[ref35] McGlynn S.A. Geiskkovitch D. Mitzner T.L. Rogers W.A. (2016). “PARO’s Stress-Reduction Potential for Older Adults.” in *Proceedings of the Human Factors and Ergonomics Society Annual Meeting September 2016*. 60, (Sage CA: Los Angeles, CA: SAGE Publications), 1799–1803.

[ref36] Meesters A. den Bosch-Meevissen Y. M. Weijzen C. A. Buurman W. A. Losen M. Schepers J.. (2018). The effect of mindfulness-based stress reduction on wound healing: a preliminary study. J. Behav. Med. 41, 385–397. doi: 10.1007/s10865-017-9901-8, PMID: 29159589

[ref37] Miller R. Kirschbaum C. (2013). “Trier social stress test,” in Encyclopedia of Behavioral Medicine, 2005–2008. eds. Gellman M. D. Turner J. R. (Springer)

[ref38] Moon H. S. Mizara A. McBride S. R. (2013). Psoriasis and psycho-dermatology. Dermatol. Ther. 3, 117–130. doi: 10.1007/s13555-013-0031-0, PMID: 24318414 PMC3889305

[ref39] Neerackal R. J. Abdul Latheef E. N. Sukumarakurup S. Jafferany M. (2020). Relaxation therapy in the management of psoriasis. Dermatol. Ther. 33:e14030. doi: 10.1111/dth.14030, PMID: 32683732

[ref40] Nomura T. Hoshina Y. (2017). “How Different Types of Animal Robots Differently Influence Elder and Younger People’s Mental States?” In *Proceedings of the Companion of the 2017 ACM/IEEE International Conference on Human-Robot Interaction March 2017*. 231–232.

[ref41] O'Leary C. J. Creamer D. Higgins E. Weinman J. (2004). Perceived stress, stress attributions and psychological distress in psoriasis. J. Psychosom. Res. 57, 465–471. doi: 10.1016/j.jpsychores.2004.03.012, PMID: 15581650

[ref42] Ouyang H. Xu J. Zhu Z. Long T. Yu C. (2015). Rapid discrimination of malignant lesions from normal gastric tissues utilizing Raman spectroscopy system: a meta-analysis. J. Cancer Res. Clin. Oncol. 141, 1835–1844. doi: 10.1007/s00432-015-1971-9, PMID: 25912559 PMC11823906

[ref43] Paradisi A. Abeni D. Finore E. Di Pietro C. Sampogna F. Mazzanti C.. (2010). Effect of written emotional disclosure interventions in persons with psoriasis undergoing narrow band ultraviolet B phototherapy. Eur. J. Dermatol. 20, 599–605. doi: 10.1684/ejd.2010.1018, PMID: 20605769

[ref44] Parisi R. Iskandar I. Y. Kontopantelis E. Augustin M. Griffiths C. E. Ashcroft D. M. (2020). National, regional, and worldwide epidemiology of psoriasis: systematic analysis and modelling study. BMJ 369:m1590. doi: 10.1136/bmj.m1590, PMID: 32467098 PMC7254147

[ref46] Piaserico S. Marinello E. Dessi A. Linder M. D. Coccarielli D. Peserico A. (2016). Efficacy of biofeedback and cognitive-behavioural therapy in psoriatic patients A single-blind, randomized and controlled study with added narrow-band ultraviolet B therapy. Acta Derm. Venereol. 96, 91–95. doi: 10.2340/00015555-2428, PMID: 27283367

[ref47] Ponsi G. Monachesi B. Panasiti V. Aglioti S. M. Panasiti M. S. (2019). Physiological and behavioral reactivity to social exclusion: a functional infrared thermal imaging study in patients with psoriasis. J. Neurophysiol. 121, 38–49. doi: 10.1152/jn.00555.2018, PMID: 30379630 PMC6383668

[ref48] Portugal-Cohen M. Horev L. Ruffer C. Schlippe G. Voss W. Oron M.. (2012). Non-invasive skin biomarkers quantification of psoriasis and atopic dermatitis: cytokines, antioxidants and psoriatic skin auto-fluorescence. Biomed. Pharmacother. 66, 293–299. doi: 10.1016/j.biopha.2011.12.009, PMID: 22397760

[ref49] Price M. L. Mottahedin I. Mayo P. R. (1991). Can psychotherapy help patients with psoriasis? Clin. Exp. Dermatol. 16, 114–117. doi: 10.1111/j.1365-2230.1991.tb00319.x, PMID: 2032371

[ref50] Qureshi A. A. Awosika O. Baruffi F. Rengifo-Pardo M. Ehrlich A. (2019). Psychological therapies in management of psoriatic skin disease: a systematic review. Am. J. Clin. Dermatol. 20, 607–624. doi: 10.1007/s40257-019-00437-7, PMID: 30937679

[ref51] Rajka G. Thune P. (1976). The relationship between the course of psoriasis and transepidermal water loss, photoelectric plethysmography and reflex photometry. Br. J. Dermatol. 94, 253–261. doi: 10.1111/j.1365-2133.1976.tb04381.x, PMID: 1252357

[ref52] Robinson H. MacDonald B. Broadbent E. (2015). Physiological effects of a companion robot on blood pressure of older people in residential care facility: a pilot study. Australas. J. Ageing 34, 27–32. doi: 10.1111/ajag.12099, PMID: 24373064

[ref53] Robinson H. MacDonald B. Kerse N. Broadbent E. (2013). The psychosocial effects of a companion robot: a randomized controlled trial. JAMDA 14, 661–667. doi: 10.1016/j.jamda.2013.02.007, PMID: 23545466

[ref54] Saito T. Shibata T. Wada K. Tanie K. (2003). “Relationship Between Interaction with the Mental Commit Robot and Change of Stress Reaction of the Elderly.” In *Proceedings 2003 IEEE International Symposium on Computational Intelligence in Robotics and Automation. Computational Intelligence in Robotics and Automation for the New Millennium July 2003*, 1, 119–124. IEEE.

[ref55] Schneider G. Heuft G. Hockmann J. (2013). Determinants of social anxiety and social avoidance in psoriasis outpatients. J. Eur. Acad. Dermatol. Venereol. 27, 383–386. doi: 10.1111/j.1468-3083.2011.04307.x, PMID: 21999164

[ref56] Shah R. Bewley A. (2014). Psoriasis: ‘the badge of shame’. A case report of a psychological intervention to reduce and potentially clear chronic skin disease. Clin. Exp. Dermatol. 39, 600–603. doi: 10.1111/ced.12339, PMID: 24758704

[ref57] Shibata T. Coughlin J. F. (2014). Trends of robot therapy with neurological therapeutic seal robot, PARO. J. Robot. Mechatron. 26, 418–425. doi: 10.20965/jrm.2014.p0418

[ref58] Sijercic I. Ennis N. Monson C. M. (2020). A systematic review of cognitive and behavioral treatments for individuals with psoriasis. J. Dermatol. Treat. 31, 631–638. doi: 10.1080/09546634.2019.1690625, PMID: 31696748

[ref59] Spitzer R. L. Kroenke K. Williams J. B. (1999). Patient health questionnaire primary care study group. Validation and utility of a self-report version of PRIME-MD: the PHQ primary care study. JAMA 282, 1737–1744. doi: 10.1001/jama.282.18.1737, PMID: 10568646

[ref60] Stewart T. J. Tong W. Whitfeld M. J. (2018). The associations between psychological stress and psoriasis: a systematic review. Int. J. Dermatol. 57, 1275–1282. doi: 10.1111/ijd.13956, PMID: 29516474

[ref61] Stone N. Stavroulaki P. Kendall C. Birchall M. Barr H. (2000). Raman spectroscopy for early detection of laryngeal malignancy: preliminary results. Laryngoscope 110, 1756–1763. doi: 10.1097/00005537-200010000-00037, PMID: 11037840

[ref62] Talari A. C. Movasaghi Z. Rehman S. Rehman I. U. (2015). Raman spectroscopy of biological tissues. Appl. Spectrosc. Rev. 50, 46–111. doi: 10.1080/05704928.2014.923902

[ref63] Tausk F. Whitmore S. E. (1999). A pilot study of hypnosis in the treatment of patients with psoriasis. Psychother. Psychosom. 68, 221–225. doi: 10.1159/000012336, PMID: 10396014

[ref64] Vargas-Obieta E. Martínez-Espinosa J. C. Martínez-Zerega B. E. Jave-Suárez L. F. Aguilar-Lemarroy A. González-Solís J. L. (2016). Breast cancer detection based on serum sample surface enhanced Raman spectroscopy. Lasers Med. Sci. 31, 1317–1324. doi: 10.1007/s10103-016-1976-x, PMID: 27289243

[ref65] Vedhara K. Morris R. M. Booth R. Horgan M. Lawrence M. Birchall N. (2007). Changes in mood predict disease activity and quality of life in patients with psoriasis following emotional disclosure. J. Psychosom. Res. 62, 611–619. doi: 10.1016/j.jpsychores.2006.12.017, PMID: 17540218

[ref66] Verhoeven E. W. Kraaimaat F. W. De Jong E. M. Schalkwijk J. Van De Kerkhof P. C. Evers A. W. (2009). Individual differences in the effect of daily stressors on psoriasis: a prospective study. Br. J. Dermatol. 161, 295–299. doi: 10.1111/j.1365-2133.2009.09194.x, PMID: 19438455

[ref67] Wada K. Shibata T. Saito T. Sakamoto K. Tanie K. (2005). “Psychological and Social Effects of One Year Robot Assisted Activity on Elderly People at a Health Service Facility for the Aged.” in *Proceedings of the 2005 IEEE International Conference on Robotics and Automation April 2005*. 2785–2790. IEEE.

[ref68] Watson D. Clark L. A. Tellegen A. (1988). Development and validation of brief measures of positive and negative affect: the PANAS scales. J. Pers. Soc. Psychol. 54, 1063–1070. doi: 10.1037/0022-3514.54.6.1063, PMID: 3397865

[ref69] Yang H. Zheng J. (2020). Influence of stress on the development of psoriasis. Clin. Exp. Dermatol. 45, 284–288. doi: 10.1111/ced.14105, PMID: 31592542

[ref70] Zachariae R. Øster H. Bjerring P. Kragballe K. (1996). Effects of psychologic intervention on psoriasis: a preliminary report. J. Am. Acad. Dermatol. 34, 1008–1015. doi: 10.1016/S0190-9622(96)90280-7, PMID: 8647966

[ref71] Zachariae R. Zachariae H. Blomqvist K. Davidsson S. Molin L. Mørk C.. (2004). Self-reported stress reactivity and psoriasis-related stress of Nordic psoriasis sufferers. J. Eur. Acad. Dermatol. Venereol. 18, 27–36. doi: 10.1111/j.1468-3083.2004.00721.x, PMID: 14678528

[ref72] Zhao J. Lui H. Kalia S. Zeng H. (2015). Real-time Raman spectroscopy for automatic in vivo skin cancer detection: an independent validation. Anal. Bioanal. Chem. 407, 8373–8379. doi: 10.1007/s00216-015-8914-9, PMID: 26231688

[ref73] Zill J. M. Christalle E. Tillenburg N. Mrowietz U. Augustin M. Härter M.. (2019). Effects of psychosocial interventions on patient-reported outcomes in patients with psoriasis: a systematic review and meta-analysis. Br. J. Dermatol. 181, 939–945. doi: 10.1111/bjd.17272, PMID: 30291741

